# Splenic autotransplantation in a patient with human immunodeficiency virus infection: a case report

**DOI:** 10.1186/1752-1947-5-379

**Published:** 2011-08-15

**Authors:** Adriana Toro, Maurizio Mannino, Giulio Reale, Isidoro Di Carlo

**Affiliations:** 1 Department of Surgical Sciences, Organ Transplantation, and Advanced Technologies, University of Catania, Cannizzaro Hospital, Via Messina 829, I-95126 Catania, Italy

## Abstract

**Introduction:**

Splenectomy is performed mostly because of traumatic events that cause rupture of the spleen. Postsplenectomy, a patient has a higher risk of developing sepsis. Autotransplantation of splenic tissue decreases the risk of opportunistic infection and sepsis, but its role in patients with human immunodeficiency virus is debated because the spleen is a replication site, especially during the asymptomatic phase of this infection. We present a case of a patient with human immunodeficiency virus infection who was admitted to our hospital for a traumatic rupture of the spleen and underwent spleen autotransplantation.

**Case presentation:**

A 36-year-old Caucasian man was admitted to the shock trauma center of our hospital after a car accident. Anamnesis showed that the patient had been human immunodeficiency virus-positive for 13 years. A computed tomography scan showed abundant fluid collection in his superior and inferior abdomen caused by splenic rupture, with no other associated intra-abdominal lesions. During surgery, the spleen appeared severely damaged. A splenectomy was performed, and 35 g of splenic tissue was autotransplanted in a pouch created in the omentum. No complications occurred after surgery, and our patient was discharged from our hospital nine days after the operation. One year later, computed tomography and scintigraphy showed that the transplanted tissue was functioning well.

**Conclusions:**

Autotransplantation of splenic tissue decreases the risk of opportunistic infection and sepsis, and it might also be useful in patients with human immunodeficiency virus. Other studies need to be done to validate this hypothesis.

## Introduction

Splenectomy is performed mostly because of traumatic events that cause rupture of the spleen [1]. Postsplenectomy, a patient is at a higher risk of developing sepsis [2]. In patients with human immunodeficiency virus (HIV) infection, a splenectomy can be performed to treat related thrombocytopenia. Autotransplantation of splenic tissue decreases the risk of opportunistic infection and sepsis, but its role in patients with HIV is debated because the spleen is a replication site, especially in the asymptomatic phase of this infection [3]. However, previous reports in the literature have shown that HIV DNA, mostly transported by CD4^+ ^T lymphocytes, can be found in the spleen. It seems that, on the basis of these studies, splenic dendritic cells can harbor the virus, but it is not likely that they can be used as a major replication site in the course of the disease [4].

HIV also directly damages splenic tissue, reducing white pulp and causing perivascular hyalinization, infarcts, necrosis and granulomatous reactions [5], but germinal centers continue to function even when infiltrated by the virus [6]. Therefore, performing a splenectomy in patients with HIV can be considered as both a positive treatment and a risk, because it eliminates a possible source of infection while weakening the already compromised immune system of these patients.

This case report discusses the case of a patient with splenic trauma that was treated successfully with autotransplantation of splenic tissue. We describe how a transplant technique involving only a small amount of splenic tissue inserted into a single pouch in the omentum can be a valid therapeutic choice in patients with HIV to balance the benefits and disadvantages of splenectomy.

## Case report

A 36-year-old Caucasian man was admitted to the shock trauma center of our hospital after a car accident. Anamnesis showed that our patient had been HIV-positive for 13 years. No other information about our patient was available at that time, because he was in a life-threatening condition and no time was available to ask for information from the infectious disease specialist engaged in treating our patient's HIV.

Laboratory tests showed an increased number of leukocytes (12.3 × 10^3^/μL; reference range, 4.1 to 10.9 × 10^3^/μL), mostly neutrophils; and high levels of lactate dehydrogenase (778 U/L; reference range, 300 to 600 U/L), creatine kinase (372 U/L; reference range, 38 to 190 U/L) and aspartate aminotransferase (63 U/L; reference range, 13 to 41 U/L). His hemoglobin, red blood cells, and hematocrit were within the reference ranges.

A computed tomography (CT) scan showed abundant fluid collection in his superior and inferior abdomen caused by splenic rupture, with no other associated intra-abdominal lesions. Surgery was acutely scheduled after we acquired our patient's informed consent. During surgery, his spleen appeared severely damaged on the surface, necrotic and extremely congested. A splenectomy was performed, and 35 g of splenic tissue was autotransplanted into a pouch created in the omentum.

No complications occurred after surgery, and our patient was discharged nine days after the operation. One year later, CT and scintigraphy showed that the transplanted tissue was functioning well.

## Discussion

Conservative treatment after splenic trauma has been suggested due to the association of sepsis and thromboembolic complications after splenectomy [7]. In patients with splenic trauma, autotransplantation is also performed in addition to the conservative treatment methods and nonoperative management.

Saving splenic tissue is important because of the role of the spleen in the immune system; asplenia is often related to a higher risk of sepsis or infections, particularly those caused by encapsulated bacteria such as *Neisseria meningitidis *or *Streptococcus pneumoniae *[8,9]. Other lymphoid tissues can respond to recurrent infections, but the spleen is fundamental in the immune response to newly encountered encapsulated bacteria. Therefore, asplenic patients are more vulnerable to new infections caused by these kinds of agents.

Autotransplantation of splenic tissue decreases the risk of opportunistic infection or sepsis, but its role in patients with HIV is debated because in people with HIV the spleen is a replication site, especially in the asymptomatic phase of the disease.

However, previous studies have demonstrated that splenectomy has a positive effect on platelet count in patients with HIV-related immune thrombocytopenia and that splenectomy has beneficial effects because the progression of HIV becomes slower than in patients with spleens [10-12]. A possible explanation for this observation is that the spleen forms approximately 50% of lymphoreticular tissue. Lymphoreticular tissue represents the major site for HIV sequestration and replication. The spleen is the ideal viral replication site, so the removal of a large proportion of lymphoreticular tissue during splenectomy may reduce the available reservoir for the HIV replication cycle. These studies, however, did not analyze the amount and the types of infections that occur during the asymptomatic phase of HIV.

Although it is pretty certain that splenic dendritic cells can harbor the virus, it is not likely that they can be used as a major replication site by the virus during the course of the disease [4].

Two explanations for the finding of HIV in germinal centers have been reported in the literature. Either the virus can arrive there after being transported on the surface of dendritic cells as an immune complex, or it can be produced in its normal place by HIV-infected antigen-specific T cells within germinal centers.

Follicular dendritic cells are exclusive structures found in the lymphoid follicles of secondary lymphoid organs. They are involved in the process of maturation of immunoglobulin-producing cells and in the process of B-cell memory, and they are not permissive for HIV infection [13]. Germinal centers, however, continue to function even when infiltrated by the virus and appear to be hyperplastic [6].

In the mouse, one of the targets of HIV is CD27^+^B220^- ^B cells, a population of cells of the peripheral blood similar to splenic marginal zone cells. Mouse models have shown that the depletion of CD27^+^B220^- ^B cells is parallel to an alteration in the splenic B-cell population caused by HIV, and this seems to be involved in the defective B-cell immunity against encapsulated bacteria such as pneumococcus [14]. Therefore, the goals of spleen autologous transplantation described in this case report were to avoid this loss of efficiency of the immune system, especially against encapsulated bacteria, and at the same time to reduce to a minimum the risk of preserving a viral reservoir by transplanting a large amount of splenic tissue. For this reason, we did not execute multiple-site transplants in the omentum as is usually reported. In fact, multiple transplants after initial necroses may develop into new tissue and become more abundant than the native spleen in the medium or long term, and this condition could increase the active replication of the virus. Therefore, we decided to transplant only 35 g of splenic tissue into an omental pouch because this quantity is effective in establishing a normal immune system and does not offer the virus a large replication site. One of the methods of performing a splenic autotransplant is to suture small pieces of splenic tissue (1-2 cm) to the greater omentum or the mesocolon, where the veins drain to the portal vein and the liver. In our patient, we transplanted a single large element with the risk of partial necrosis to avoid the risk of viral replication in multiple elements [15].

As previously discussed, the spleen is the ideal replication site for HIV, but the choice to reimplant splenic tissue should be given high regard, considering that after the operation the patient will have far less tissue than with an entire spleen, so the risk of restoring a good site for major viral replication is drastically reduced.

Splenic autotransplantation affords clinicians the possibility of maintaining the marginal zone, which is an essential part of the spleen involved in the response to encapsulated bacteria and in rapid humoral response to blood-borne antigens [16]. Therefore, performing autotransplantation is useful in building a response to bacteria during a period when HIV does not attack T-cell function and marginal zone function too much. In this way, autotransplantation has the advantage of making use of an eventual secondary response during periods that may follow when immune deficiency becomes important.

## Conclusion

Autotransplantation of splenic tissue decreases the risk of opportunistic infection and sepsis, and its role should be useful in patients with HIV. Other studies are needed to validate this hypothesis.

## Consent

Written informed consent was obtained from the patient for publication of this case report and any accompanying images. A copy of the written consent is available for review by the Editor-in-Chief of this journal.

## Competing interests

The authors declare that they have no competing interests.

## Authors' contributions

AT developed the study concept and design. MM and GR acquired the data. AT, MM and GR performed the analysis and interpretation of data. AT drafted the manuscript. IDC did critical revision of the manuscript. IDC supervised the study.

All authors have read and approved the final manuscript.
